# Comparative Evaluation of the Age, Intake, Weight, and Walking Tool and Common Nutrition-Screening Tools in Patients with Oral Squamous Cell Carcinoma: An Ambispective Cohort Study

**DOI:** 10.1016/j.cdnut.2026.109562

**Published:** 2026-10-24

**Authors:** Yiming Zhang, Yu Huang, Hanchen Zhou, Xue Zhang, Hao Wang, Chang Liu, Jinzhong Li, Ming Su, Chong Wang, Zhengxue Han, Hanping Shi, Zhien Feng

**Affiliations:** 1 Department of Oral and Maxillofacial-Head and Neck Oncology, Beijing Stomatological Hospital, Capital Medical University, Beijing, China; 2 Department of Clinical Nutrition, Capital Medical University, Beijing, China; 3 Department of Gastrointestinal Surgery/Department of Clinical Nutrition, Beijing Shijitan Hospital, Capital Medical University, Beijing, China

**Keywords:** nutrition screening, oral squamous cell carcinoma, malnutrition, PG-SGA, NRS-2002, MUST

## Abstract

**Background:**

Malnutrition is highly prevalent in patients with oral squamous cell carcinoma (OSCC) and adversely affects clinical outcomes, yet a standardized nutritional screening tool for this population remains lacking.

**Objectives:**

This study evaluated the agreement and association of the Age, Intake, Weight, and Walking (AIWW) tool with established nutritional screening and assessment tools, as well as its prognostic value, in patients with OSCC.

**Methods:**

A total of 422 patients with OSCC (254 males and 168 females; mean age 61.17 ± 12.09 y) between 2021 and 2024 were enrolled in the ambispective cohort study. Patient-Generated Subjective Global Assessment (PG-SGA) classifications were used as a common comparator for pairwise agreement analyses. Pairwise comparisons included overall agreement, positive and negative agreement, Cohen’s κ, Gwet’s first-order agreement coefficient, and Kendall’s τ-b coefficient. Survival analysis was performed using the Kaplan–Meier analyses with log-rank tests and Cox regression models.

**Results:**

The prevalence of nutritional risk ranged from 24.9% to 66.6% across different screening tools. At the PG-SGA ≥4 threshold, overall agreement was 70.1% for AIWW, 79.4% for Nutritional Risk Screening 2002 (NRS-2002), and 77.7% for Malnutrition Universal Screening Tool (MUST); positive agreement was 72.4%, 70.9%, and 66.4%, respectively. Kendall’s τ-b coefficient was 0.494, 0.576, and 0.550, respectively. High nutritional risk identified by AIWW and MUST was independently associated with poorer overall survival (*P* < 0.050). AIWW (Brier score: 0.096; C-statistic: 0.677, 95% confidence interval: 0.609, 0.740) had favorable apparent predictive performance for overall survival.

**Conclusions:**

AIWW shows moderate agreement with PG-SGA classifications and identifies a larger proportion of patients as nutritionally at risk than NRS-2002 or MUST. Its high-risk classification is independently associated with poorer survival. These findings support AIWW as a preliminary screening option, whereas discordant classifications require followed by comprehensive nutritional assessment.

This trial was registered at Chinese Clinical Trial Registry as ChiCTR2400088053 (https://www.chictr.org.cn/showprojEN.html?proj=233591).

## Introduction

Oral squamous cell carcinoma (OSCC) is the most prevalent malignancy of the oral and maxillofacial region and the 16th most common cancer worldwide. It presents significant clinical challenges because of its unique anatomical location at the beginning of the digestive tract [1], [2]. It often induces severe dysphagia, odynophagia, and mechanical obstruction, which compromise nutritional intake [3]. Consequently, malnutrition affects ≤40% of patients with OSCC [4]. Despite advances in multimodal therapy, including surgery, radiotherapy [5], and chemotherapy [6], survival outcomes remain poor. Five-year overall survival (OS) is ∼64%, and disease-free survival (DFS) is ∼79% [7]. Preoperative malnutrition is associated with increased surgical complications, prolonged hospitalization, reduced treatment tolerance, deteriorated quality of life, and reduced OS [8], [9], [10], [11]. Thus, developing and implementing an effective, simple nutritional screening method for patients with OSCC are essential.

Nutritional risk screening and assessment tools are critical for the early identification and management of malnutrition. Widely implemented screening tools include the Nutritional Risk Screening 2002 (NRS-2002) and the Malnutrition Universal Screening Tool (MUST) [12], [13]. Assessment tools include the Patient-Generated Subjective Global Assessment (PG-SGA) and the Global Leadership Initiative on Malnutrition (GLIM) criteria [14], [15], [16]. PG-SGA is an internationally used, cancer-specific nutritional assessment instrument with extensive validation [17], [18], [19], [20].

Recently, the Age, Intake, Weight, and Walking (AIWW) tool has emerged as a novel screening tool specifically designed for patients with cancer [21]. Initial studies encompassed patients with lung, colorectal, and breast cancers, reporting promising screening utility. Findings suggest that AIWW demonstrates high sensitivity in patients with cancer. This high sensitivity is particularly valuable for screening, where missed identification of nutritional risk should be minimized [22]. However, AIWW has not been validated in patients with head and neck cancer, raising questions about its performance in this distinct population with unique nutritional challenges.

Several studies have compared different nutritional screening tools against PG-SGA-assessed malnutrition as the comparative criteria in various types of cancers [19], [23]. In a cohort of patients with head and neck squamous cell carcinoma (HNSCC), 28% of the patients were at nutritional risk according to NRS-2002, and 34% were diagnosed as malnutrition by PG-SGA [24]. Similarly, MUST identified 31.3% of patients with HNSCC as being at risk of malnutrition [25]. Another study included 6 common nutritional screening tools to predict malnutrition in older adults. The researchers found that MUST was the most valid tool for evaluating nutritional risk, whereas NRS-2002 tended to overestimate risk [26]. These studies did not provide consistent, satisfactory screening efficacy; therefore, AIWW warrants further evaluation.

This study therefore aimed to compare the agreement and association of AIWW classifications with PG-SGA status alongside NRS-2002 and MUST, and to evaluate the prognostic associations of each screening tool in an OSCC cohort, trying to provide evidence supporting an optimized nutritional screening approach for this population.

## Methods

### Study population

Between September 2021 and December 2024, we conducted an ambispective cohort study of 598 patients with OSCC at the Department of Oral and Maxillofacial-Head and Neck Oncology, Beijing Stomatological Hospital, Capital Medical University. The ambispective design comprised data from the Prospective, Observational, Real-world Oral Malignant Tumors Study database. On the basis of the ethics approval timeline, data from September 2021 to December 2023 were extracted from the database to form a retrospective cohort, whereas data from January 2024 to December 2024 served as the prospective cohort. This study fully complied with recognized ethical principles, including the World Medical Association Declaration of Helsinki (2002 version), and was approved by the Institutional Review Board of Beijing Stomatological Hospital of Capital Medical University (approval number: CMUSH-IRB-KJ-PJ-2024-29). Written informed consent was obtained from all participants before enrollment.

The inclusion criteria were as follows: *1*) histologically confirmed, newly diagnosed OSCC without prior treatment; *2*) scheduled to undergo curative surgery for OSCC, without neoadjuvant chemotherapy or radiotherapy; *3*) aged ≥18 y; and *4*) no distant metastasis before treatment. The exclusion criteria were as follows: *1*) treatment refusal or poor general condition; *2*) insufficient compliance; *3*) missing follow-up data; and *4*) missing data necessary for nutritional screening and assessment. A total of 176 patients were excluded based on these criteria, and 422 patients were eventually enrolled in this study (Figure 1). Among the excluded patients, 119 patients had no follow-up data, 42 had incomplete information required for PG-SGA, NRS-2002, MUST, or AIWW classification, and 15 had no weight-loss data. The retrospective and prospective cohorts included 294 and 128 patients, respectively. All patients were followed for ≥12 mo.

**FIGURE 1:**
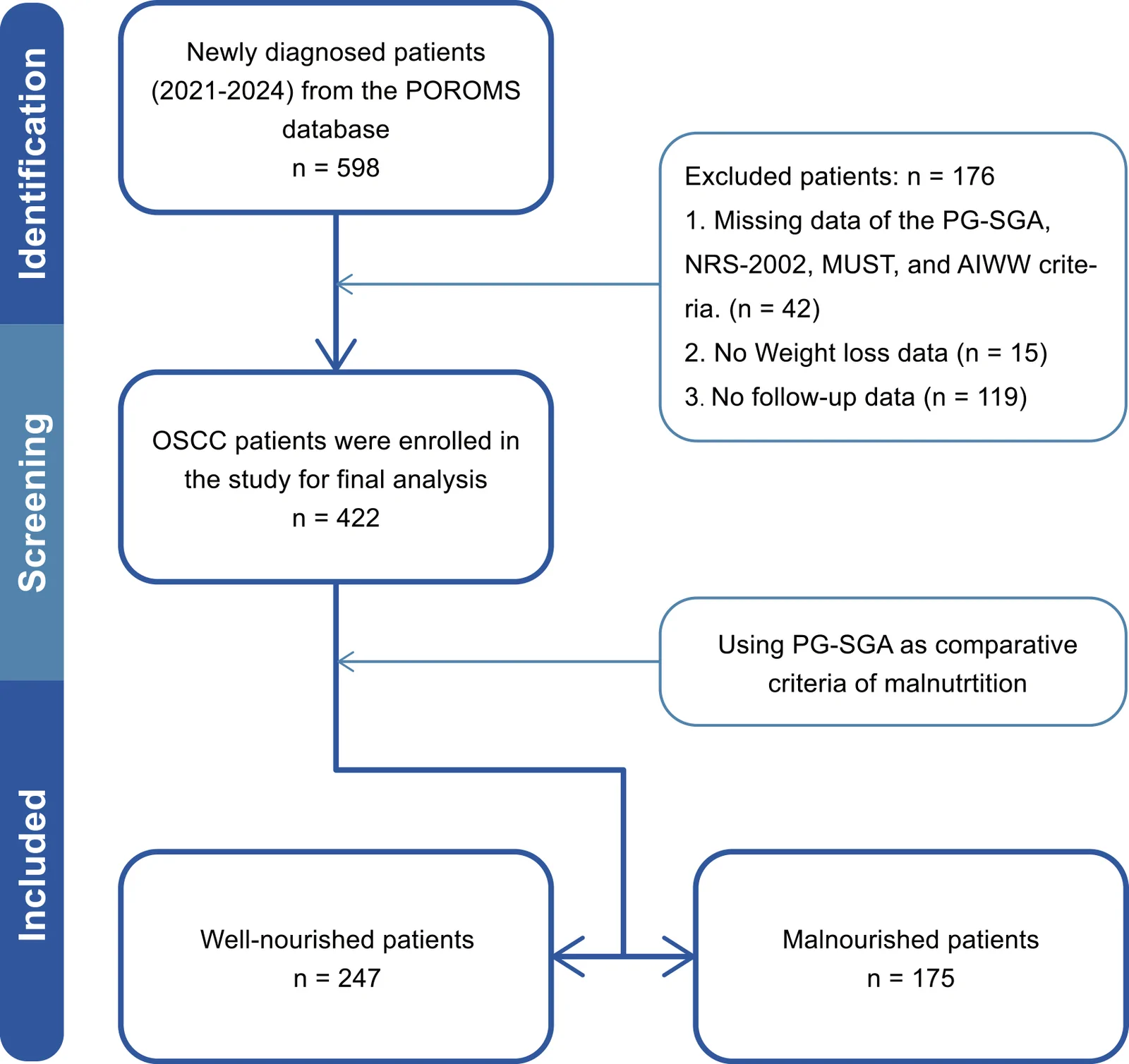
Flow chart of the present study. AIWW, Age, Intake, Weight, and Walking tool; MUST, Malnutrition Universal Screening Tool; NRS-2002, Nutritional Risk Screening 2002; OSCC, oral squamous cell carcinoma; POROMS, Prospective, Observational, Real-world Oral Malignant Tumors Study.

The sample size was calculated as follows: using the Schoenfeld sample size formula, with a 2-sided *α* = 0.05 and *β* = 0.1, an expected hazard ratio of 2.0 (nutritional risk compared with no risk), and an estimated nutritional risk of *P* = 0.40. Assuming a clinical outcome event rate of 25%, the minimum required sample size to achieve adequate statistical power was 364 patients. Considering a potential 15% attrition rate due to loss of follow-up, noncompliance, or unexpected death, a total of 422 participants were planned for inclusion.

### Data collection

Professional training was provided to doctors who collected patients’ clinical and nutrition-related information within 24 h of hospital admission before curative surgery. All nutritional screening tools (AIWW, NRS-2002, and MUST) were applied contemporaneously during both retrospective and prospective data collection. Baseline data included general, nutritional, and anthropometric variables. General variables included age, sex, clinical and pathological tumor stage, and pathological grade. Nutritional variables included reduced appetite or food intake, unintentional decrease in walking ability, and involuntary weight loss. Anthropometric variables included height, weight, waist, and hip circumference. On the basis of acquired data, BMI was calculated, where BMI = weight/(height)^2^.

### Research endpoints

Prognostic indicators included OS and DFS. OS was defined as the time interval between the date of the initial surgery and the patient’s death. DFS was defined as the time from the date of the first operation until the first documented recurrence, metastasis, or death from any cause. Follow-up was conducted through outpatient visits and telephone interviews scheduled every 3 mo and 6 mo, respectively. The cutoff date for follow-up was December 2025.

### Nutritional screening and assessment methods

According to the AIWW criteria (Table 1), malnutrition risk was defined as a score of ≥1 on the 4 questions; each question scores 1 point for an answer of yes and 0 points for a no. To distinguish different levels of nutritional risk, a validated threshold score of ≥2 was used to define high nutritional risk [21].

**TABLE 1:** The AIWW questionnaire

No.	Contents	Questions	Answer
Q1	Age	Are you over 65 y old?	No/Yes
Q2	Intake	Have you noticed a decrease in appetite or food intake in the past month?	No/Yes
Q3	Weight	Did you lose your weight involuntarily over the past month?	No/Yes
Q4	Walking	Did your walking pace, walking steps, or walking distance decrease involuntarily over the past month?	No/Yes

Abbreviation: AIWW, Age, Intake, Weight, and Walking.

The MUST was developed to assess the risk of protein-energy undernutrition [12]. It includes 3 parameters scored 0, 1, or 2 based on severity as follows: *1*) BMI >20 kg/m^2^ = 0; 18.5 to 20.0 kg/m^2^ = 1; <18.5 kg/m^2^ = 2; *2*) unintentional weight loss in the past 3 to 6 mo <5% = 0; 5% to 10% = 1; >10% = 2; *3*) abrosia >5 d absent = 0; present = 2. Overall risk of malnutrition is evaluated by the sum of 3 parts: 0 refers to low risk, 1 refers to medium nutritional risk, and ≥2 refers to high risk, respectively.

NRS-2002 is a nutritional screening tool proposed to pinpoint patients who would benefit from nutritional intervention [13]. It comprises 3 components: *1*) disease severity score: 1 to 3 points refer to regional and systemic diseases. *2*) Nutritional condition score: 1 point for weight loss >5% in the past 3 mo or food intake 50% to 75% of normal in the recent week; 2 points for weight loss >5% in the past 2 mo or BMI 18.5 to 20.5 kg/m^2^ or food intake 25% to 50% of normal; 3 points for weight loss >5% in 1 mo, >15% in 3 mo, or BMI <18.5 kg/m^2^, ALB <35 g/L, or food intake reduced to 0% to 25% compared with the normal condition. *3*) Age score: 1 point for patients ≥70 y. The total NRS-2002 score is the sum of these 3 subsections. A total score of ≥3 indicates high risk and warrants nutritional support.

PG-SGA is a widely used assessment tool that is well established for both malnutrition assessment and prognostic evaluation [14]. The PG-SGA consists of 2 parts: the first part surveys recent dietary intake, weight loss, symptoms, activities, and function; the second part captures disease specifics and metabolic demands. Each item is scored and summed: those who score <4 were defined as well nourished (stage 1), scores of 4 to 8 were defined as stage 2, and scores ≥9 were defined as stage 3.

### Statistical analysis

Statistical analysis was performed using IBM SPSS Statistics version 29.0.2. Graphs and diagrams were generated by OriginPro 2021 software for Windows and R software (version 4.5.3; R Core Team). For continuous variables, they were presented as means and SDs, or medians and IQRs. Categorical variables were summarized as absolute and relative frequencies. The significance of differences between 2 groups was evaluated by Pearson’s chi-squared test and Fisher’s exact test (categorical variables) or an independent *t*-test (continuous variables). Taking PG-SGA as the comparative criteria, Cohen’s κ statistics were calculated to ascertain classification agreement between the AIWW criteria and other common nutrition-screening tools. κ ≥ 0.75 indicates high consistency, κ between 0.4 and 0.75 indicates moderate consistency, and κ < 0.4 represents poor consistency.

Pairwise classification comparisons treated PG-SGA as a common comparator rather than as a comparative criterion. Overall agreement was the proportion of concordant pairs. Positive agreement was calculated as 2*a*/(2*a*+*b*+*c*), and negative agreement as 2*d*/(2*d*+*b*+*c*), where *a* and *d* represented both-positive and both-negative pairs, and *b* and *c* represented the 2 discordant parts [27]. Cohen’s κ and Gwet’s first-order agreement coefficient (Gwet’s AC1) quantified chance-corrected agreement, and Kendall’s τ-b quantified ordinal association. Bias-corrected and accelerated 95% confidence intervals (CIs) for agreement estimates were obtained using 5000 patient-level bootstrap resamples. All tests were 2-sided, and *P* < 0.05 was considered statistically significant.

### Survival analysis

Kaplan–Meier survival curves were generated according to nutritional status, and differences in outcomes among these groups were assessed via the log-rank test. The reverse Kaplan–Meier method was applied to calculate the median follow-up time.

Cox proportional-hazards regression models were used to identify factors associated with survival outcomes in patients with OSCC. The proportional hazards assumption was evaluated using Schoenfeld residuals, revealing no significant deviation from proportionality (*P* > 0.050). Univariable Cox analysis evaluated potential prognostic factors, including age, sex, smoking, drinking, diabetes, T stage, N stage, tumor site, comorbidities, and nutritional assessment parameters. For the multivariable analysis, NRS-2002 (binary; 1 degree of freedom), MUST (3 levels; 2 degrees of freedom), and AIWW (3 levels; 2 degrees of freedom) were entered into separate models with the same specification and fitted for OS and DFS. Sex and age were adjusted in both models, resulting in 7 degrees of freedom in total. Hazard ratios (HRs) and corresponding 95% CIs were calculated to quantify the association between each factor and survival outcomes. The predictive performance of separate Cox regression models for each screening tool was evaluated using the C-statistic and Brier score. A higher predictive accuracy of the model refers to a C-statistic close to 1 and a Brier score close to 0. The discriminative performance was evaluated by time-dependent receiver operating characteristic (ROC). Prespecified clinical timepoints were set at 1 and 3 y after surgery, and the area under the time-dependent ROC curve (time-dependent AUC) was calculated for each tool at these time points.

## Results

### Patient characteristics

In total, 422 participants (254 males and 168 females, aged 61.17 ± 12.09 y) were enrolled in the present study. The median follow-up time was 26 mo. As shown in Table 2, baseline characteristics were stratified by nutritional status according to PG-SGA. The tongue was the most common primary tumor site (45.5%, 192/422). The classification of T stages and N stages indicated that 142 patients had T stage ≥3, whereas the majority of patients had T2 stage tumors (144, 34.1%). Lymph node metastasis was observed in 111 patients (26.3%). Patients with N1, N2, and N3 stages numbered 68 (16.1%), 38 (9.0%), and 5 (1.2%), respectively.

**TABLE 2:** Detailed baseline characteristics of the 422 patients enrolled according to PG-SGA

Characteristic^1^	Overall	Well-nourished	Malnutrition	*P* value⁎
	*n* = 422	*n* = 247	*n* = 175	
Sex				0.502
Male	254 (60.2)	152 (61.5)	102 (58.3)	
Female	168 (39.8)	95 (38.5)	73 (41.7)	
BMI (kg/m^2^)				<0.001
≥18.5	402 (95.3)	245 (99.2)	157 (89.7)	
<18.5	20 (4.7)	2 (0.8)	18 (10.3)	
Age (y)				0.011
<65	231 (54.7)	148 (59.9)	83 (47.4)	
≥65	191 (45.3)	99 (40.1)	92 (52.6)	
Smoking				0.494
Yes	194 (46.0)	117 (47.4)	77 (44.0)	
No	228 (54.0)	130 (52.6)	98 (56.0)	
Drinking				0.478
Yes	153 (36.3)	93 (37.7)	60 (34.3)	
No	269 (63.7)	154 (62.3)	115 (65.7)	
Diabetes				0.519
Yes	90 (21.3)	50 (20.2)	40 (22.9)	
No	332 (78.7)	197 (79.8)	135 (77.1)	
T stage				<0.001
T1	136 (32.3)	95 (38.5)	41 (23.4)	
T2	144 (34.1)	94 (38.1)	50 (28.6)	
T3	41 (9.7)	19 (7.7)	22 (12.6)	
T4a	82 (19.4)	33 (13.4)	49 (28.0)	
T4b	19 (4.5)	6 (2.4)	13 (7.4)	
N stage				0.006
N0	311 (73.7)	196 (79.4)	115 (65.7)	
N1	68 (16.1)	33 (13.4)	35 (20.0)	
N2	38 (9.0)	18 (7.3)	20 (11.4)	
N3	5 (1.2)	0 (0.0)	5 (2.9)	
Tumor site				0.481
Tongue	192 (45.5)	104 (42.1)	88 (50.3)	
Buccal	63 (14.9)	43 (17.4)	20 (11.4)	
Gingiva	71 (16.8)	41 (16.6)	30 (17.1)	
Hard palate	10 (2.4)	6 (2.4)	4 (2.3)	
Floor of mouth	31 (7.3)	20 (8.1)	11 (6.3)	
Others	55 (13.0)	33 (13.4)	22 (12.6)	

Abbreviation: PG-SGA, Patient-Generated Subjective Global Assessment.

1 Values are presented as proportions *n* (%) for categorical variables.

⁎ Significance of comparisons between categorical variables was carried out using a chi-square test, with Fisher correction if necessary.

According to the AIWW criteria, 35.8% (151/422) and 30.8% (130/422) of the patients were classified as having moderate and high nutritional risk, respectively. Moreover, the number of patients with AIWW scores of 0 to 4 were 141 (33.4%), 151 (35.8%), 78 (18.5%), 40 (9.5%), and 12 (2.8%), respectively. A total of 175 (41.5%) patients were defined as malnourished by PG-SGA, and the prevalence of nutritional risk was highest for AIWW (66.6%), followed by NRS-2002 (29.4%) and MUST (24.9%). Other demographic and nutritional characteristics are presented in Supplemental Tables 1 to 4.

### Agreement analyses

Using the PG-SGA ≥4 as the reference of malnutrition, overall agreement was 70.1% (95% CI: 65.6%, 74.6%) for AIWW, 79.4% (95% CI: 75.4%, 82.9%) for NRS-2002, and 77.7% (95% CI: 73.7%, 81.5%) for MUST. Positive agreement was 72.4% (95% CI: 67.3%, 76.8%), 70.9% (95% CI: 64.8%, 76.6%), and 66.4% (95% CI: 60.0%, 72.6%), respectively; negative agreement was 67.5% (95% CI: 62.1%, 72.6%), 84.0% (95% CI: 80.6%, 87.1%), and 83.3% (95% CI: 79.6%, 86.4%), respectively. Using PG-SGA ≥9 as the reference of severely malnourished, overall agreement was 83.6%, 84.1%, and 86.7%; positive agreement was 64.2%, 64.2%, and 46.2%; and negative agreement was 89.4%, 89.8%, and 92.4% for AIWW, NRS-2002, and MUST, respectively (Table 3).

**TABLE 3:** Statistical evaluation of nutritional screening tools using PG-SGA as comparative criteria

Characteristics^1^	AIWW	NRS-2002	MUST
Malnutrition (PG-SGA ≥4)			
Overall agreement, % (95% CI)	70.1 (65.6, 74.6)	79.4 (75.4, 82.9)	77.7 (73.7, 81.5)
Positive agreement, % (95% CI)	72.4 (67.3, 76.8)	70.9 (64.8, 76.6)	66.4 (60.0, 72.6)
Negative agreement, % (95% CI)	67.5 (62.1, 72.6)	84.0 (80.6, 87.1)	83.3 (79.6, 86.4)
Gwet’s AC1 (95% CI)	0.407 (0.313, 0.488)	0.620 (0.539, 0.689)	0.600 (0.518, 0.672)
Cohen’s kappa (κ)	0.435 (0.362, 0.511)⁎⁎	0.556 (0.475, 0.634)⁎⁎	0.513 (0.428, 0.587)⁎⁎
Kendall’s tau coefficient	0.494	0.576	0.550
Severely malnourished (PG-SGA ≥9)			
Overall agreement, % (95% CI)	83.6 (79.9, 87.0)	84.1 (80.6, 87.4)	86.7 (83.2, 89.6)
Positive agreement, % (95% CI)	64.2 (56.1, 71.7)	64.2 (55.8, 72.2)	46.2 (34.3, 58.4)
Negative agreement, % (95% CI)	89.4 (86.7, 91.7)	89.8 (87.2, 92.1)	92.4 (90.2, 94.2)
Gwet’s AC1 (95% CI)	0.747 (0.682, 0.805)	0.758 (0.693, 0.812)	0.831 (0.779, 0.871)
Cohen’s kappa (κ)	0.552 (0.464, 0.638)⁎⁎	0.553 (0.463, 0.645)⁎⁎	0.390 (0.262, 0.520)⁎⁎
Kendall’s tau coefficient	0.613	0.422	0.401

For comparisons with PG-SGA score ≥4, positive classifications were AIWW ≥1, NRS-2002 ≥3, and MUST ≥1. For comparisons with PG-SGA score ≥9, positive classifications were AIWW ≥2, NRS-2002 ≥3, and MUST ≥2.

Abbreviations: AIWW, Age, Intake, Weight, and Walking Tool; CI, confidence interval; MUST, Malnutrition Universal Screening Tool; NRS-2002, Nutritional Risk Screening 2002; PG-SGA, Patient-Generated Subjective Global Assessment.

1 Values are presented as proportions *n* (lower 95% CI, upper 95% CI).

⁎⁎ *P* < 0.01.

At PG-SGA ≥4, Cohen’s κ values were 0.435 (95% CI: 0.362, 0.511) for AIWW, 0.556 (95% CI: 0.475, 0.634) for NRS-2002, and 0.513 (95% CI: 0.428, 0.587) for MUST; the corresponding Gwet’s AC1 values were 0.407 (95% CI: 0.313, 0.488), 0.620 (95% CI: 0.539, 0.689), and 0.600 (95% CI: 0.518, 0.672). Other statistical results at PG-SGA ≥9 are shown in Table 3.

### Prognosis

In total, the numbers of patients who experienced OS and DFS events were 60 and 114, respectively. Kaplan–Meier analysis showed that nutritional risk defined by the AIWW, NRS-2002, and MUST was associated with OS (log-rank *P* < 0.050). Only AIWW was significantly associated with DFS (log-rank *P* < 0.050), whereas NRS-2002 (*P* = 0.240) and MUST (*P* = 0.121) were not (Figure 2).

**FIGURE 2:**
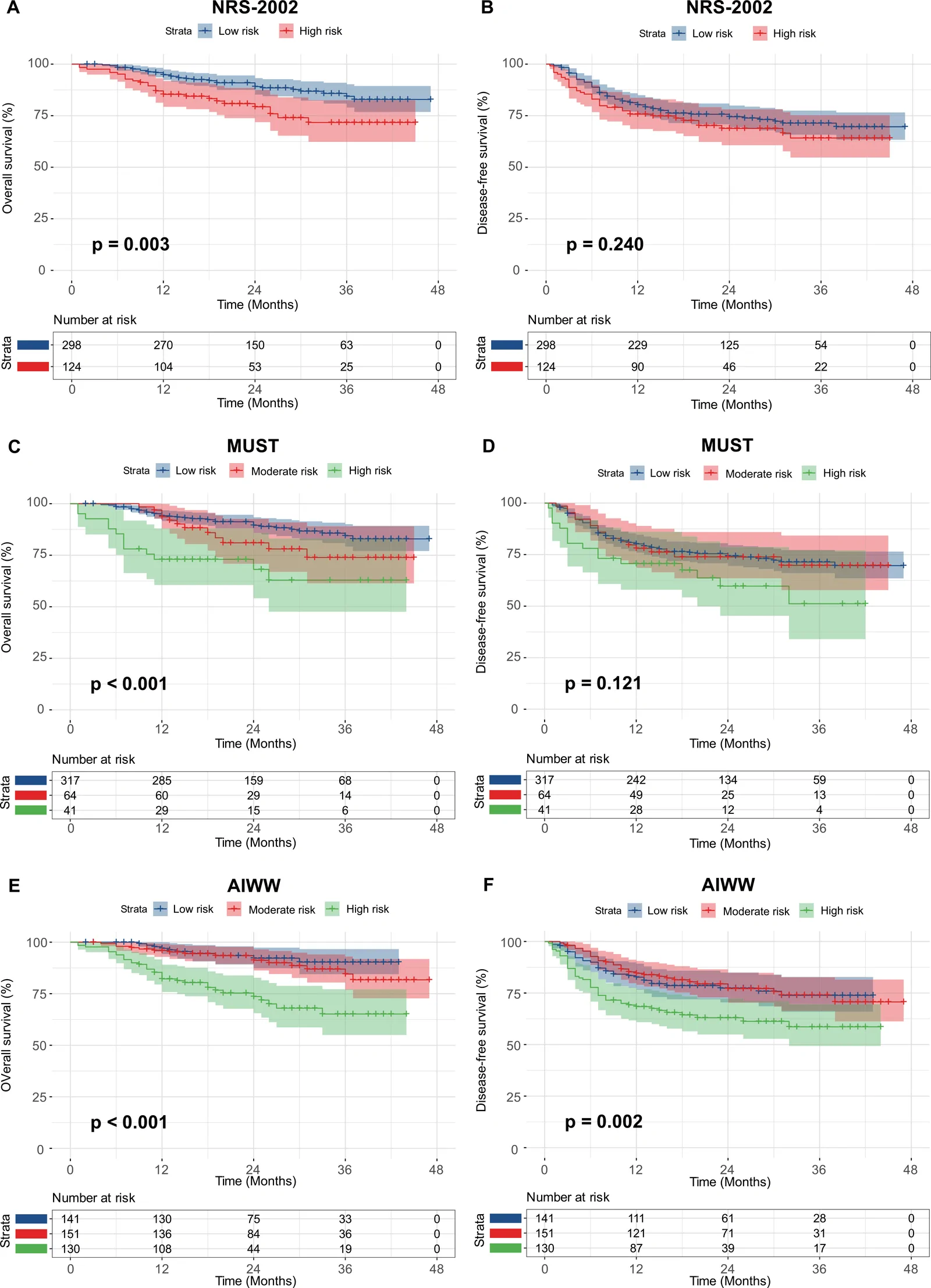
Kaplan–Meier curves for overall survival and disease-free survival according to nutritional risk defined by each screening tool. (A, B) Overall survival and disease-free survival for NRS-2002. (C, D) Overall survival and disease-free survival for MUST. (E, F) Overall survival and disease-free survival for AIWW. AIWW, Age, Intake, Weight, and Walking tool; MUST, Malnutrition Universal Screening Tool; NRS-2002, Nutritional Risk Screening 2002.

Nutritional risk identified by multiple criteria was significantly associated with an increased risk of mortality. Univariable Cox regression analysis revealed that according to PG-SGA, moderately (HR = 2.06, 95% CI: 1.09, 3.89; *P* = 0.027) and severely malnourished (HR = 5.50, 95% CI: 3.00, 10.11; *P* < 0.001) were associated with higher mortality risk. High nutritional risk defined by the AIWW (HR = 4.479, 95% CI: 2.209, 9.081; *P* < 0.001), NRS-2002 (HR = 2.116, 95% CI: 1.272, 3.520; *P* = 0.004), and MUST (HR = 3.580, 95% CI: 1.893, 6.770; *P* < 0.001) was also significantly associated with OS (Figure 3).

**FIGURE 3:**
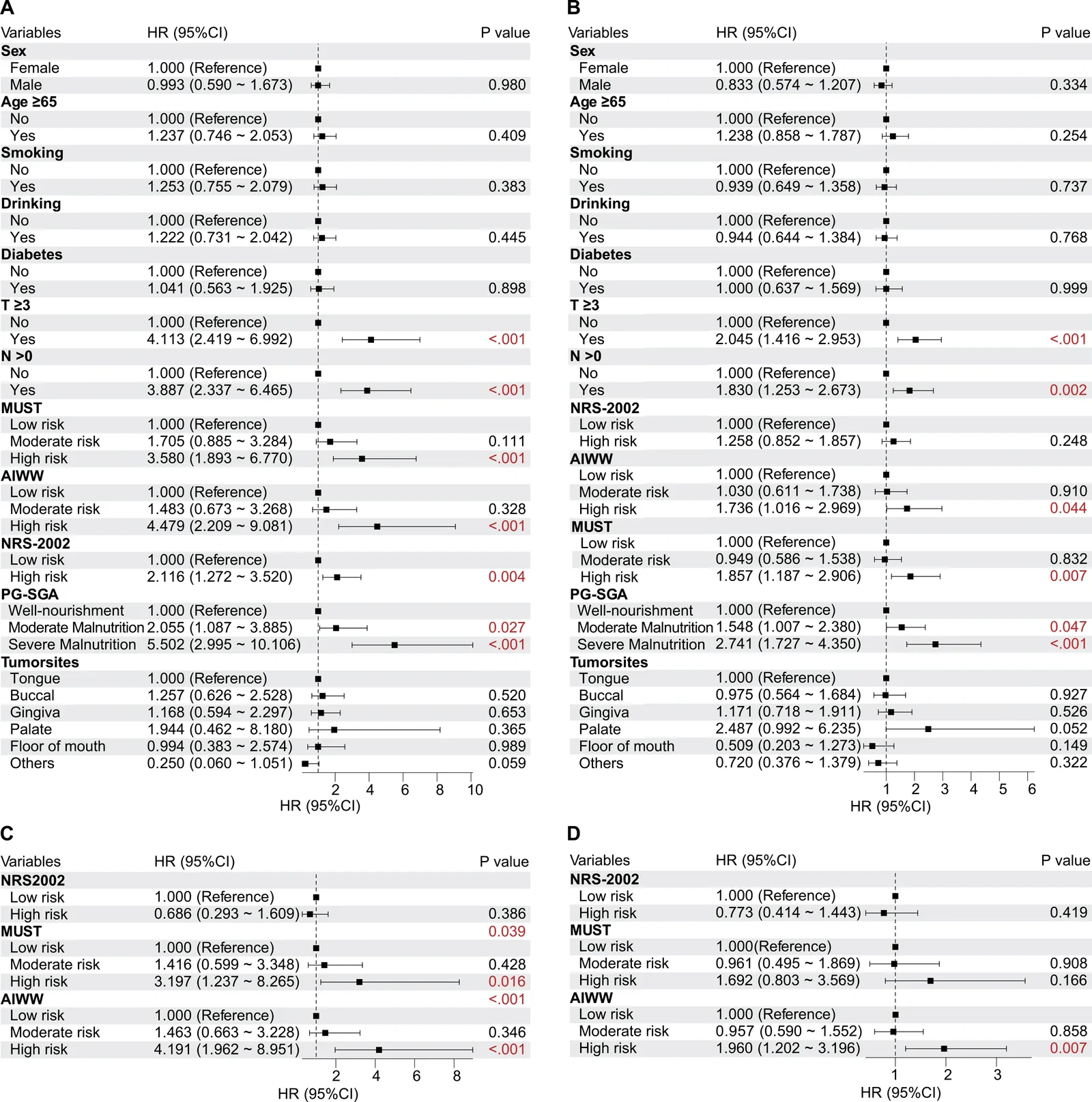
Univariable and multivariable Cox regression analyses of overall survival (OS) and disease-free survival (DFS) in patients with OSCC. (A) Univariable regression analysis of OS. (B) Univariable regression analysis of DFS. (C) Multivariable regression analysis of OS. (D) Multivariable regression analysis of DFS. Both multivariable OS and DFS models were adjusted for sex and age. AIWW, Age, Intake, Weight, and Walking tool; CI, confidence interval; HR, hazard ratio; MUST, Malnutrition Universal Screening Tool; NRS-2002, Nutritional Risk Screening 2002; OSCC, oral squamous cell carcinoma; PG-SGA, Patient-Generated Subjective Global Assessment.

Moderately malnourished (HR = 1.55, 95% CI: 1.01, 2.38; *P* = 0.047) and severely malnourished (HR = 2.74, 95% CI: 1.73, 4.35; *P* < 0.001) as defined by PG-SGA, as well as high nutritional risk of the AIWW (HR = 1.736, 95% CI: 1.016, 2.969; *P* = 0.044) and MUST (HR = 1.857, 95% CI: 1.187, 2.906; *P* = 0.007) were significantly associated with DFS.

Multivariable regression analyses (Figure 3) were adjusted for sex and age in both models. Collinearity testing showed that the variance inflation factors for NRS-2002, MUST, and AIWW classifications were <3, indicating no significant collinearity. The models contained 7 degrees of freedom with 5 parameters. With 60 OS events and 114 DFS events, the events-per-variable ratios were 8.6 and 16.3, respectively. High nutritional risk as defined by MUST (HR = 3.197, 95% CI: 1.237, 8.265; *P* = 0.016) and AIWW (HR = 4.191, 95% CI: 1.962, 8.951; *P* < 0.001) were independent prognostic factors of OS. Only high nutritional risk as defined by AIWW (HR = 1.960, 95% CI: 1.202, 3.196; *P* = 0.007) demonstrated as an independent prognostic factor of DFS.

For predictive performance of OS, AIWW showed a Brier score of 0.096 and a C-statistic of 0.677 (95% CI: 0.609, 0.740). Brier scores of MUST and NRS-2002 were 0.099 and 0.101, respectively. C-statistics were 0.617 (95% CI: 0.545, 0.686) and 0.599 (95% CI: 0.534, 0.673) for MUST and NRS-2002, respectively. For DFS, the Brier scores were 0.180, 0.184, and 0.185 for AIWW, MUST, and NRS-2002, respectively. C-statistics were 0.577 (95% CI: 0.518, 0.630) for AIWW, 0.530 (95% CI: 0.483, 0.573) for MUST, and 0.528 (95% CI: 0.479, 0.573) for NRS-2002.

At 12 mo, the time-dependent AUCs for OS were 0.671 (95% CI: 0.608, 0.735), 0.604 (95% CI: 0.542, 0.669), and 0.587 (95% CI: 0.526, 0.656) for AIWW, MUST, and NRS-2002, respectively. At 36 mo, the corresponding AUCs were 0.665 (95% CI: 0.602, 0.732), 0.597 (95% CI: 0.537, 0.659), and 0.585 (95% CI: 0.522, 0.650) for AIWW, MUST, and NRS-2002, respectively.

## Discussion

Preoperative nutritional risk represents a critical feature of patients with cancer and serves as an important prognostic determinant [28]. Nutritional screening is widely recognized as the foundation for nutritional assessment and subsequent intervention [29], [30], [31]. From a clinical perspective, the novel screening tool AIWW offers advantages in simplicity, usability, and ease of interpretation, requires minimal training, and may be suitable for self-administration. However, the applicability of AIWW in nutritional risk screening for oral cancer remains unclear. Therefore, this study aimed to evaluate its agreement and association with established screening and assessment tools, together with its prognostic associations. A previous study demonstrated the AIWW’s robust screening efficacy and prognostic predictive value [21]. This study represents the first validation of AIWW specifically in patients with OSCC. Its higher positive agreement facilitates clear identification of mild and moderate nutritional risk, providing empirical support for targeted nutritional screening in the OSCC population.

In this study, 29.4% (124/422) and 24.9% (105/422) of patients were classified as at nutritional risk by NRS-2002 and MUST, respectively. A higher proportion (281/422, 66.6%) of patients were identified as at nutritional risk by AIWW. AIWW prioritized positive agreement over negative agreement in this OSCC cohort. The higher proportion of discordant positive classifications refers to the fact that more patients with latent nutritional risk are categorized as under AIWW nutritional risk, while reducing the possibility of omission for patients who are actually malnourished as defined by PG-SGA. From another perspective, the broader positive classification and lower negative agreement of AIWW may also introduce redundant comprehensive assessment to patients.

A systematic review of 21 studies reported sensitivities and specificities for MUST compared with PG-SGA of 0.84 (95% CI: 0.73, 0.91) and 0.85 (95% CI: 0.75, 0.91), respectively; corresponding values for NRS-2002 were 0.76 (95% CI: 0.58, 0.87) and 0.86 (95% CI: 0.76, 0.93) [32]. AIWW, which showed a different agreement profile characterized by higher positive agreement and lower negative agreement than NRS-2002 and MUST, may be particularly useful when minimizing missed cases is prioritized. The overlap in assessment domains in weight change, intake, and body function of PG-SGA and AIWW may contribute to this positive agreement. De Groot et al. [33] evaluated GLIM-defined malnutrition against the PG-SGA in ambulatory patients with cancer. Using the PG-SGA as the comparator, the GLIM criteria yielded a sensitivity of 76% and a specificity of 73%. Collectively, these results demonstrate a different agreement profile of AIWW, although Cohen’s κ and Gwet’s AC1 were numerically lower than NRS-2002 and MUST.

Unintentional weight loss is a major clinical concern in patients with cancer. Recent studies have demonstrated that weight loss is linked to increased all-cause mortality and may serve as an independent prognostic factor in patients with advanced OSCC undergoing surgery [34], [35]. In this study, 132 of 422 patients (31.3%) experienced involuntary weight loss. Among these patients, 90% (119/132) were classified as malnourished according to PG-SGA. Vandersteen et al. [36] reported that weight loss >10% was significantly associated with postoperative fistula formation and wound infection in patients with head and neck cancers (HNC) undergoing microvascular reconstruction. By incorporating the recent 1-mo unintentional weight loss, AIWW can identify more patients at risk and may facilitate early detection of nutritional imbalance and support timely intervention. This potential benefit requires further validation.

The results of our study further indicated that patients with high nutritional risk had more advanced tumor progression, with a proportion of *T* ≥ 3 reaching 48.0% compared with 23.5% in the low-risk group. In addition, 43.1% of patients with malnutrition reported severe pain or trismus, compared with 12.8% in well-nourished patients. These findings suggest that impaired nutrient intake because of local tumor progression may contribute to nutritional deterioration. As widely established in the literature, advanced tumor-node-metastasis classification (TNM stages) of OSCC are independently associated with lower survival rates [30], [37]. A vicious cycle between tumor progression and worsening nutritional status may underlie these observations.

The prognostic power of AIWW was primarily driven by patients with high nutritional risk (HR = 4.479; 95% CI: 2.209, 9.081; *P* < 0.001), but not moderate risk (HR = 1.483; 95% CI: 0.673, 3.268; *P* = 0.328). As for OS, a lower Brier score of 0.096 and a higher C-statistic of 0.677 (95% CI: 0.609, 0.740) than MUST and NRS-2002 indicated that AIWW had a better apparent predictive performance. After adjustment for sex and age, the multivariable regression model showed that high nutritional risk defined by either AIWW (HR = 4.191; 95% CI: 1.962, 8.951; *P* < 0.001) and MUST (HR = 3.197; 95% CI: 1.237, 8.265; *P* = 0.016) remained as independent prognostic factors for OS, whereas NRS-2002 did not show a significant association. Consistent with our findings, the study published by Gomes-Neto et al. [19] revealed that only high malnutrition risk defined by MUST correlated with a higher risk of 6-mo mortality after adjustment for age, sex, hospital ward, and hospitalization. The comparable prognostic performance of AIWW and MUST may be attributed to shared screening components, namely, intake insufficiency and involuntary weight loss. Although the unintended weight loss plays an essential role in cancer cachexia, representing dysregulated metabolism, poor treatment tolerance, and increased comorbidity burden [38]. A retrospective study including 242 patients with HNC illustrated that having a MUST score of ≥2 was significantly associated with postoperative complications [25]. Given the clinical goals of personalized nutritional intervention and prognostic prediction, further prospective and multicenter evaluation is warranted before AIWW can be recommended as a potential screening option in the OSCC population.

Several limitations should be acknowledged in our study. First, this was a single-center study with a relatively short median follow-up (26 mo). Second, we compared AIWW only with NRS-2002 and MUST criteria using PG-SGA as a reference method, although other screening tools are available. Unfortunately, although the GLIM criteria are a diagnostic framework that has been widely validated, the limited anthropometric data prevented retrospective application of the GLIM criteria, which reduced the methodological strength of the nutritional status comparison. Third, the PG-SGA is a subjective nutritional assessment tool whose initial component relies on patient self-report, which may therefore introduce recall bias. The lower negative agreement of AIWW at PG-SGA ≥4 and its broader positive classification may increase referrals for comprehensive assessment among patients whose PG-SGA scores remain below the threshold. This clinical burden should be evaluated prospectively. The multivariable models were mutually adjusted for the 3 nutritional screening tools but did not include demographic or tumor-related clinical covariates except for sex and age; therefore, residual confounding cannot be excluded. Model complexity was restricted because only 60 OS events were observed. Further studies with longer follow-up, diversified nutritional screening and assessment tools, and multicenter data are warranted to improve the reliability of our conclusions.

In conclusion, to our knowledge, this is the first study to evaluate the applicability of the AIWW within a single tumor type. As defined by the AIWW criteria, the prevalence of moderate and high nutritional risk is ≤35.8% and 30.8% among patients with OSCC in this study. AIWW shows substantial agreement with PG-SGA, and its high-risk classification is independently associated with OS. This study highlights both differences and concordance among commonly used nutritional screening tools and suggests that AIWW offers a simple and convenient screening approach based on subjective information alone. Further multicenter studies are needed to validate AIWW in larger and more diverse OSCC populations.

## Author contributions

The authors’ responsibilities were as follows **–** YMZ, JZL, MS, CW, ZXH, HPS, ZEF: designed the research; YMZ, YH, HCZ, XZ, HW, CL: conducted the research; YMZ, YH: analyzed the data; YMZ: wrote the manuscript; HPS, ZEF: took primary responsibility for the final content; and all authors: read and approved the final manuscript.

## Data Availability

Data described in the manuscript, code book, and analytic code will be made available upon request pending application and approval.

## Declaration of generative AI and AI-assisted technologies in the writing process

The authors declare that no generative AI or AI-assisted technologies were used in the writing of this manuscript.

## Funding

This work was supported by The Beijing High-Level Innovation and Entrepreneurship Talent Support Program Young Top Talent Projects (no. G202522151), the National Natural Science Foundation of China (no. 82573934), the Natural Science Foundation of Beijing Municipality (no. 7252058), and the Capital′s Funds for Health Improvement and Research (CFH-2026-2-2142). The supporting source had no involvement or restrictions regarding publication.

## Conflict of interest

The authors report no conflicts of interest.
