# Supplementary material for: Comparative Evaluation of the Age, Intake, Weight, and Walking Tool and Common Nutrition-Screening Tools in Patients with Oral Squamous Cell Carcinoma: An Ambispective Cohort Study
Source: Curr Dev Nutr. 2026 Oct 24;10(11):109562. doi: 10.1016/j.cdnut.2026.109562 (PMC13770589; doi:10.1016/j.cdnut.2026.109562)
Supplement: Supplementary file 1 [file mmc1.docx]

Supplementary Material

**Comparative evaluation of the Age, Intake, Weight, and Walking (AIWW) Tool and common nutrition-screening tools in patients with oral squamous cell carcinoma: An ambispective cohort study**

Yiming Zhang ^1^, Yu Huang ^1^, Hanchen Zhou ^1^, Xue Zhang ^1^, Hao Wang ^1^, Chang Liu ^1^, Jinzhong Li ^1^, Ming Su ^1^, Chong Wang ^1^, Zhengxue Han ^1^, Hanping Shi ^2,3^,*, Zhien Feng ^1, 2^,*

^1^ Department of Oral and Maxillofacial-Head and Neck Oncology, Beijing Stomatological Hospital, Capital Medical University, Beijing 100070, China.

^2^ Department of Clinical Nutrition, Capital Medical University, Beijing 100069, China.

^3^ Department of Gastrointestinal Surgery/Department of Clinical Nutrition, Beijing Shijitan Hospital, Capital Medical University, Beijing 100038, China.

*Correspondence:

Hanping Shi, M.D., Ph.D. Department of Gastrointestinal Surgery/Department of Clinical Nutrition, Beijing Shijitan Hospital, Capital Medical University, Beijing 100038, China; Email: shihp@ccmu.edu.cn

Zhien Feng, M.D., Ph.D., Department of Oral and Maxillofacial-Head and Neck Oncology, Beijing Stomatological Hospital, Capital Medical University, No. 9 Fanjiacun Road, Fengtai District; Department of Clinical Nutrition, Capital Medical University, Beijing 100070, P.R. China. Phone: 86-10-57099963. Fax: 86-10-57099963. Email: jyfzhen@mail.ccmu.edu.cn

**Abbreviations:**

AIWW: Age, Intake, Weight, and Walking Tool

BMI: Body Mass Index

CI: Confidence Interval

DFS: Disease-free Survival

GLIM: Global Leadership Initiative on Malnutrition

Gwet’s AC1: Gwet’s first-order agreement coefficient

HNC: Head and Neck Cancer

HNSCC: Head and Neck Squamous Cell Carcinoma

HR: Hazard Ratio

MUST: Malnutrition Universal Screening Tool

NRS-2002: Nutritional Risk Screening 2002

OS: Overall Survival

OSCC: Oral Squamous Cell Carcinoma

PG-SGA: Patient-Generated Subjective Global Assessment

POROMS: Prospective, Observational, Real-world Oral Malignant Tumors Study

**Table S1.** Detailed baseline characteristics of the study population based on PG-SGA.

| **Characteristic ^a^** | **Overall** | **Well-nourished** | **Malnutrition** | ***p* value** |
| --- | --- | --- | --- | --- |
|  | N=422 | N=247 | N=175 |  |
| Sex |  |  |  | 0.501 |
| Male | 254 (60.2) | 152 (61.5) | 102 (58.3) |  |
| Female | 168 (39.8) | 95 (38.5) | 73 (41.7) |  |
| Age, year | 61.17 (12.09) | 60.81 (11.60) | 61.68 (12.71) | <0.001 |
| BMI, kg/m^2^ | 24.59 (4.02) | 25.32 (3.74) | 23.55 (4.16) | <0.001 |
| BMI, kg/m^2^ |  |  |  | <0.001 |
| ≥18.5 | 402 (95.3) | 245 (99.2) | 157 (89.7) |  |
| <18.5 | 20 (4.7) | 2 (0.8) | 18 (10.3) |  |
| Age, year |  |  |  | 0.011 |
| <65 | 231 (54.7) | 148 (59.9) | 83 (47.4) |  |
| ≥65 | 191 (45.3) | 99 (40.1) | 92 (52.6) |  |
| Reduce food intake |  |  |  | <0.001 |
| No | 309 (73.2) | 242 (98.0) | 67 (38.3) |  |
| Yes | 113 (26.8) | 5 (2.0) | 108 (61.7) |  |
| Lost weight in past month |  |  |  | <0.001 |
| No | 290 (68.7) | 234 (94.7) | 56 (32.0) |  |
| Yes | 132 (31.3) | 13 (5.3) | 119 (68.0) |  |
| Diminish of physical function |  |  |  | <0.001 |
| No | 383 (90.8) | 236 (95.5) | 147 (84.0) |  |
| Yes | 39 (9.2) | 11 (4.5) | 28 (16.0) |  |
| AIWW score ^b^ |  |  |  | <0.001 |
| 0 | 141 (33.4) | 131 (53.0) | 10 (5.7) |  |
| 1 | 151 (35.8) | 104 (42.1) | 47 (26.9) |  |
| 2 | 78 (17.5) | 12 (4.9) | 66 (37.7) |  |
| 3 | 40 (9.5) | 0 (0.0) | 40 (22.9) |  |
| 4 | 12 (2.8) | 0 (0.0) | 12 (6.9) |  |
| AIWW risk ^b^ |  |  |  | <0.001 |
| No | 141 (33.4) | 131 (53.0) | 10 (5.7) |  |
| Yes | 281 (66.6) | 116 (47.0) | 165 (94.3) |  |
| NRS-2002 score ^b^ |  |  |  | <0.001 |
| 1 | 196 (46.4) | 166 (67.2) | 30 (17.1) |  |
| 2 | 102 (24.2) | 63 (25.5) | 39 (22.3) |  |
| 3 | 47 (11.1) | 15 (6.1) | 32 (18.3) |  |
| 4 | 65 (15.4) | 3 (1.2) | 62 (35.4) |  |
| 5 | 12 (2.8) | 0 (0.0) | 12 (6.9) |  |
| 6 | 0 (0.0) | 0 (0.0) | 0 (0.0) |  |
| NRS-2002 risk ^b^ |  |  |  | <0.001 |
| No | 298 (70.6) | 229 (92.7) | 69 (39.4) |  |
| Yes | 124 (29.4) | 18 (7.3) | 106 (60.6) |  |
| PG-SGA score ^b^ | 4.19 (3.58) | 1.77 (0.76) | 7.61 (3.18) | <0.001 |
| PG-SGA stage ^b^ |  |  |  | <0.001 |
| 1 | 247 (58.5) | 247 (100.0) | 0 (0.0) |  |
| 2 | 112 (26.5) | 0 (0.0) | 112 (64.0) |  |
| 3 | 63 (14.9) | 0 (0.0) | 63 (36.0) |  |
| Malnutrition (PG-SGA) |  |  |  | <0.001 |
| No | 247 (58.5) | 247 (100.0) | 0 (0.0) |  |
| Yes | 175 (39.3) | 0 (0.0) | 175 (100.0) |  |
| MUST risk ^b^ |  |  |  | <0.001 |
| No | 317 (75.1) | 235 (95.1) | 82 (46.9) |  |
| Yes | 105 (24.9) | 12 (4.9) | 93 (53.1) |  |
| MUST stage ^b^ |  |  |  | <0.001 |
| 0 | 317 (75.1) | 235 (95.1) | 82 (46.9) |  |
| 1 | 64 (15.2) | 10 (4.0) | 54 (30.9) |  |
| 2 | 41 (9.7) | 2 (0.8) | 39 (22.3) |  |

^a^ For continuous variables, values are presented as the mean (standard deviation, SD), or median [interquartile range, IQR], and as number (proportions) for categorical variables.

^b^ PG-SGA: The Scored Patient-Generated Subjective Global Assessment; NRS-2002: Nutrition Risk Screening 2002; LOPHS: Length of postoperative hospital stay; AIWW: A nutrition-screening tool named age, intake, weight, and walking; MUST: Malnutrition Universal Screening Tool.

Malnutrition was defined as a PG-SGA score ≥ 4, severe malnutrition was defined as a PG-SGA score ≥ 9. Malnutrition risk of NRS-2002 was defined as NRS-2002 score ≥ 3. Malnutrition risk and severe malnutrition risk of AIWW was defined as AIWW score ≥ 1 and score ≥ 2. MUST stages were defined as low risk: score = 0, moderate risk: score = 1, high risk: score ≥ 2.

**Table S2.** Detailed characteristics of AIWW.

| **Characteristic ^a^** | **AIWW ^b^** | | |
| --- | --- | --- | --- |
|  | **no risk** | **risk** | ***p* value** |
|  | 141 | 281 |  |
| Sex |  |  | 0.058 |
| Male | 94 (66.7) | 160 (56.9) |  |
| Female | 47 (33.3) | 121 (43.1) |  |
| Age, year | 53.65 (8.84) | 64.95 (11.71) | <0.001 |
| BMI, kg/m^2^ | 25.07 (4.23) | 24.35 (3.88) | <0.001 |
| BMI, kg/m^2^ |  |  | 0.414 |
| ≥18.5 | 136 (96.5) | 266 (94.7) |  |
| <18.5 | 5 (3.5) | 15 (5.3) |  |
| Age, year |  |  | <0.001 |
| <65 | 141 (100.0) | 90 (32.0) |  |
| ≥65 | 0 (0.0) | 191 (68.0) |  |
| Reduce food intake | | | <0.001 |
| No | 141 (100.0) | 168 (59.8) |  |
| Yes | 0 (0.0) | 113 (40.2) |  |
| Lost weight in past month | | | <0.001 |
| No | 141 (100.0) | 149 (53.0) |  |
| Yes | 0 (0.0) | 132 (47.0) |  |
| Diminish of physical function | | | <0.001 |
| No | 141 (100.0) | 242 (86.1) |  |
| Yes | 0 (0.0) | 39 (13.9) |  |
| NRS-2002 score ^b^ |  |  | <0.001 |
| 1 | 118 (83.7) | 78 (27.8) |  |
| 2 | 5 (3.5) | 97 (34.5) |  |
| 3 | 12 (8.5) | 35 (12.5) |  |
| 4 | 6 (4.3) | 59 (21.0) |  |
| 5 | 0 (0.0) | 12 (4.3) |  |
| 6 | 0 (0.0) | 0 (0.0) |  |
| NRS-2002 risk ^b^ |  |  | <0.001 |
| No | 123 (87.2) | 175 (62.3) |  |
| Yes | 18 (12.8) | 106 (37.7) |  |
| PG-SGA score ^b^ | 1.54 (0.99) | 5.53 (3.66) | <0.001 |
| PG-SGA stage ^b^ |  |  | <0.001 |
| 1 | 131 (92.9) | 116 (41.3) |  |
| 2 | 10 (7.1) | 102 (36.3) |  |
| 3 | 0 (0.0) | 63 (22.4) |  |
| Malnutrition (PG-SGA) |  |  | <0.001 |
| No | 131 (92.9) | 116 (41.3) |  |
| Yes | 10 (7.1) | 165 (58.7) |  |
| MUST risk ^b^ |  |  | <0.001 |
| No | 124 (87.9) | 193 (68.7) |  |
| Yes | 17 (12.1) | 88 (31.3) |  |
| MUST stage ^b^ |  |  | <0.001 |
| 0 | 124 (87.9) | 193 (68.7) |  |
| 1 | 10 (7.1) | 54 (19.2) |  |
| 2 | 7 (5.0) | 34 (12.1) |  |

^a^ For continuous variables, values are presented as the mean (standard deviation, SD), or median [interquartile range, IQR], and as number (proportions) for categorical variables.

^b^ PG-SGA: The Scored Patient-Generated Subjective Global Assessment; NRS-2002: Nutrition Risk Screening 2002; LOPHS: Length of postoperative hospital stay; AIWW: A nutrition-screening tool named age, intake, weight, and walking; MUST: Malnutrition Universal Screening Tool.

Malnutrition was defined as PG-SGA score ≥ 4, severe malnutrition was defined as a PG-SGA score ≥ 9. Malnutrition risk of NRS-2002 was defined as NRS-2002 score ≥ 3. Malnutrition risk and severe malnutrition risk of AIWW was defined as AIWW score ≥ 1 and score ≥ 2. MUST stages were defined as low risk: score = 0, moderate risk: score = 1, high risk: score ≥ 2.

**Table S3.** Detailed characteristics of NRS-2002.

| **Characteristic ^a^** | **NRS-2002 ^b^** | | |
| --- | --- | --- | --- |
|  | **no risk** | **risk** | ***p* value** |
|  | 298 | 124 |  |
| Sex |  |  | 0.463 |
| Male | 176 (59.1) | 78 (62.9) |  |
| Female | 122 (40.9) | 46 (37.1) |  |
| Age, year | 60.95 (11.98) | 61.70 (12.30) |  |
| BMI, kg/m^2^ | 25.69 (3.49) | 21.95 (3.97) | <0.001 |
| BMI, kg/m^2^ |  |  | <0.001 |
| ≥18.5 | 298 (100.0) | 104 (83.9) |  |
| <18.5 | 0 (0.0) | 20 (16.1) |  |
| Age, year |  |  | 0.687 |
| <65 | 165 (55.4) | 66 (53.2) |  |
| ≥65 | 133 (44.6) | 58 (46.8) |  |
| Reduce food intake |  |  | <0.001 |
| No | 260 (87.2) | 49 (39.5) |  |
| Yes | 38 (12.8) | 75 (60.5) |  |
| Lost weight in past month |  |  | <0.001 |
| No | 249 (83.6) | 41 (33.1) |  |
| Yes | 49 (16.4) | 83 (66.9) |  |
| Diminish of physical function |  |  | <0.001 |
| No | 281 (94.3) | 102 (82.3) |  |
| Yes | 17 (5.7) | 22 (17.7) |  |
| AIWW score ^b^ |  |  | <0.001 |
| 0 | 123 (41.3) | 18 (14.5) |  |
| 1 | 126 (42.3) | 25 (20.2) |  |
| 2 | 36 (12.1) | 42 (33.9) |  |
| 3 | 13 (4.4) | 27 (21.8) |  |
| 4 | 0 (0.0) | 12 (19.7) |  |
| AIWW risk ^b^ |  |  | <0.001 |
| No | 123 (41.3) | 18 (14.5) |  |
| Yes | 175 (58.7) | 106 (85.5) |  |
| PG-SGA score ^b^ | 2.59 (1.85) | 8.04 (3.80) | <0.001 |
| PG-SGA stage ^b^ |  |  | <0.001 |
| 1 | 229 (76.8) | 18 (14.5) |  |
| 2 | 66 (22.1) | 46 (37.1) |  |
| 3 | 3 (1.0) | 60 (48.4) |  |
| Malnutrition (PG-SGA) |  |  | <0.001 |
| No | 229 (76.8) | 18 (14.5) |  |
| Yes | 69 (23.2) | 106 (85.5) |  |
| MUST risk ^b^ |  |  | <0.001 |
| No | 285 (95.6) | 32 (25.8) |  |
| Yes | 13 (4.4) | 92 (74.2) |  |
| MUST stage ^b^ |  |  | <0.001 |
| 0 | 285 (95.6) | 32 (25.8) |  |
| 1 | 12 (4.0) | 52 (41.9) |  |
| 2 | 1 (0.3) | 40 (32.3) |  |

^a^ For continuous variables, values are presented as the mean (standard deviation, SD), or median [interquartile range, IQR], and as number (proportions) for categorical variables.

^b^ PG-SGA: The Scored Patient-Generated Subjective Global Assessment; NRS-2002: Nutrition Risk Screening 2002; LOPHS: Length of postoperative hospital stay; AIWW: A nutrition-screening tool named age, intake, weight, and walking; MUST: Malnutrition Universal Screening Tool.

Malnutrition was defined as PG-SGA score ≥ 4, severe malnutrition was defined as a PG-SGA score ≥ 9. Malnutrition risk of NRS-2002 was defined as NRS-2002 score ≥ 3. Malnutrition risk and severe malnutrition risk of AIWW was defined as AIWW score ≥ 1 and score ≥ 2. MUST stages were defined as low risk: score = 0, moderate risk: score = 1, high risk: score ≥ 2.

**Table S4.** Detailed characteristics of MUST

| **Characteristic ^a^** | **MUST ^b^** | | |
| --- | --- | --- | --- |
|  | **no risk** | **risk** | ***p* value** |
|  | 317 | 105 |  |
| Sex |  |  | 0.182 |
| Male | 185 (58.4) | 69 (65.7) |  |
| Female | 132 (41.6) | 36 (34.3) |  |
| Age, year | 61.84 (11.65) | 59.17 (13.09) | <0.001 |
| BMI, kg/m^2^ | 25.40 (3.62) | 22.15 (4.15) | <0.001 |
| BMI, kg/m^2^ |  |  | <0.001 |
| ≥18.5 | 317 (100.0) | 85 (81.0) |  |
| <18.5 | 0 (0.0) | 20 (19.0) |  |
| Age, year |  |  | 0.211 |
| <65 | 168 (53.0) | 63 (60.0) |  |
| ≥65 | 149 (47.0) | 42 (40.0) |  |
| Reduce food intake |  |  | <0.001 |
| No | 261 (82.3) | 48 (45.7) |  |
| Yes | 56 (17.7) | 57 (53.8) |  |
| Lost weight in past month |  |  | <0.001 |
| No | 255 (80.4) | 35 (33.3) |  |
| Yes | 62 (19.6) | 70 (66.7) |  |
| Diminish of physical function |  |  | 0.029 |
| No | 294 (92.7) | 89 (84.8) |  |
| Yes | 23 (7.3) | 16 (15.2) |  |
| AIWW score ^b^ |  |  | <0.001 |
| 0 | 124 (39.1) | 17 (16.2) |  |
| 1 | 127 (40.1) | 24 (22.9) |  |
| 2 | 41 (12.9) | 37 (35.2) |  |
| 3 | 19 (6.0) | 21 (20.0) |  |
| 4 | 6 (1.9) | 6 (5.7) |  |
| AIWW risk ^b^ |  |  | <0.001 |
| No | 124 (39.1) | 17 (16.2) |  |
| Yes | 193 (60.9) | 88 (83.8) |  |
| NRS-2002 score ^b^ |  |  | <0.001 |
| 1 | 191 (60.3) | 5 (4.8) |  |
| 2 | 94 (29.7) | 8 (7.8) |  |
| 3 | 25 (7.9) | 22 (21.0) |  |
| 4 | 7 (2.2) | 58 (55.2) |  |
| 5 | 0 (0.0) | 12 (11.4) |  |
| 6 | 0 (0.0) | 0 (0.0) |  |
| NRS-2002 risk ^b^ |  |  | <0.001 |
| No | 285 (89.9) | 13 (12.4) |  |
| Yes | 32 (10.1) | 92 (87.6) |  |
| PG-SGA score ^b^ | 2.88 (2.28) | 8.17 (3.83) | <0.001 |
| PG-SGA stage ^b^ |  |  | <0.001 |
| 1 | 235 (74.1) | 12 (11.4) |  |
| 2 | 71 (22.4) | 41 (39.0) |  |
| 3 | 11 (3.5) | 52 (49.5) |  |
| Malnutrition (PG-SGA) |  |  | <0.001 |
| No | 235 (74.1) | 12 (11.4) |  |
| Yes | 82 (25.9) | 93 (88.6) |  |

^a^ For continuous variables, values are presented as the mean (standard deviation, SD), or median [interquartile range, IQR], and as number (proportions) for categorical variables.

^b^ PG-SGA: The Scored Patient-Generated Subjective Global Assessment; NRS-2002: Nutrition Risk Screening 2002; LOPHS: Length of postoperative hospital stay; AIWW: A nutrition-screening tool named age, intake, weight, and walking; MUST: Malnutrition Universal Screening Tool.

Malnutrition was defined as PG-SGA score ≥ 4, severe malnutrition was defined as a PG-SGA score ≥ 9. Malnutrition risk of NRS-2002 was defined as NRS-2002 score ≥ 3. Malnutrition risk and severe malnutrition risk of AIWW was defined as AIWW score ≥ 1 and score ≥ 2. MUST stages were defined as low risk: score = 0, moderate risk: score = 1, high risk: score ≥ 2.
